# *In Vitro* Anti-*Candida* Activity of Certain New 3-(1*H*-Imidazol-1-yl)propan-1-one Oxime Esters

**DOI:** 10.3390/molecules181012208

**Published:** 2013-09-30

**Authors:** Mohamed I. Attia, Azza S. Zakaria, Maha S. Almutairi, Soraya W. Ghoneim

**Affiliations:** 1 Department of Pharmaceutical Chemistry, College of Pharmacy, King Saud University, Riyadh 11451, Saudi Arabia; 2 Medicinal and Pharmaceutical Chemistry Department, Pharmaceutical and Drug Industries Research Division, National Research Centre, Dokki, Giza 12622, Egypt; 3 Department of Pharmaceutics, College of Pharmacy, King Saud University, Riyadh 11451, Saudi Arabia; 4 Department of Microbiology, Faculty of Pharmacy, Alexandria University, Alexandria 21500, Egypt

**Keywords:** synthesis, Mannich reaction, azoles, oxime esters, anti-*Candida*

## Abstract

Anti-*Candida* activities of certain new oximes **4a**–**d** and their respective aromatic esters **5a**–**l** are reported. The tested compounds **4a**–**d** and **5a**–**l** exhibited better anti-*Candida* profiles than fluconazole. Compound **5j**, namely (*E*)-3-(1*H*-imidazol-1-yl)-1-phenylpropan-1-one *O*-4-chlorobenzoyl oxime emerged as the most active congener, with a MIC value of 0.0054 µmol/mL being more potent than both fluconazole (MIC > 1.6325 µmol/mL) and miconazole (MIC value = 0.0188 µmol/mL) as a new anti-*Candida albicans* agent.

## 1. Introduction

Fungal infections have recently emerged as a growing threat to human health, especially in patients with weakened or compromised immune systems [1,2]. The organisms most often responsible for invasive fungal infection are *Candida* and *Aspergillus* species [3]. *Candida* infections are adverse in their manifestations, varying from superficial skin problems, chronic infection of the nails, mouth, throat or vagina to frequently fatal systemic diseases that involve the lungs, heart, gastrointestinal tract, central nervous system and other organs [4]. These infections are considered to be opportunistic in nature, since some aspect of the host’s defense system is impaired in some way. In spite of the large number of the available antifungal agents, the medical need is still largely unmet and therefore, efforts to discover new antifungal agents are a must. This is largely due to the perceived threat of emerging new pathogenic fungi and resistance of many strains to existing therapy [5,6,7].

**Figure 1:**
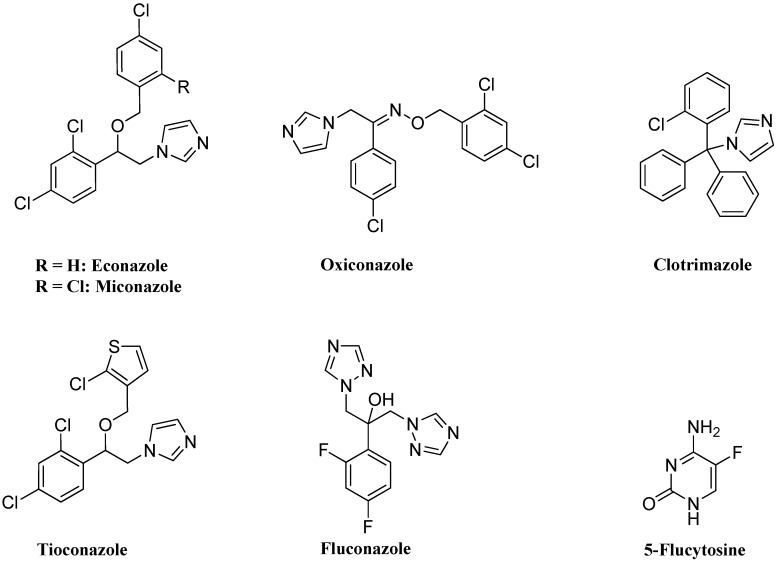
Azole antifungal agents used in clinical therapy.

Five major classes of the clinically used antifungal drugs are available, namely polyenes (such as amphotericin B and nystatin), echinocandins (such as caspofungin), allylamines (such as naftifine and terbinafine), fluoropyrimidines (such as 5-fluorocytosine) and azoles (such as miconazole, fluconazole and oxiconazole) (Figure 1) [8,9,10]. Azole antifungal drugs remain the mainstay of therapy for candidal life-threatening fungal infections due to their safety profile and high therapeutic index [11]. The mechanism of action of azole antifungals relies on their ability to inhibit Cyt-P450 dependent sterol 14α-demethylase through binding to the heme cofactor of the cytochrome CYP51 leading to inhibition of sterols synthesis in fungi [4,12].

An evaluation of the literature exposed that some potent clinically used azole antifungals are derived from oxime-containing scaffolds [13]. Additionally, most of the available imidazole-containing antifungal agents have a two carbon atom spacer between the imidazole pharmacophore and an aromatic moiety, whereas limited information is available about imidazole-containing antifungals having a three-carbon atom linker between the imidazole pharmacophore and the aromatic moiety [14,15]. Moreover, Walker *et al.* reported that some aryl and aralkyl esters of 2-(1*H*-imidazol-1-yl)-1-phenylethanols displayed more anti-*Candida albicans* activity than miconazole [16].

Based upon the aforementioned premises, we became interested in the development of new imidazole-containing drug-like anti-*Candida* agents incorporating oxime functionality, exemplified by compounds **4a**–**d** as well as their respective aromatic esters, compounds **5a**–**l**.

## 2. Results and Discussion

### 2.1. Chemistry

**Scheme 1:**
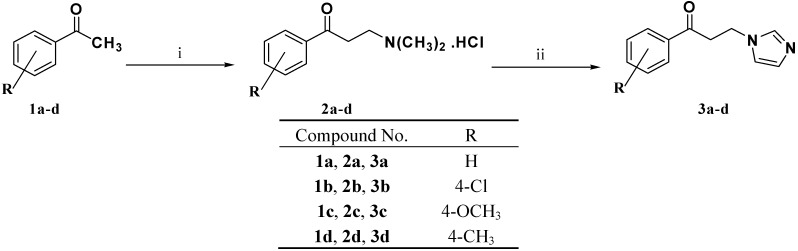
Synthesis of the ketones **3a**–**d**.

The pivotal ketones **3a**–**d** were prepared using the synthetic strategy outlined in Scheme 1. Thus, the appropriate acetophenone **1a**–**d** was reacted with dimethylamine hydrochloride and paraformaldehyde in the presence of a catalytic amount of concentrated hydrochloric acid to yield Mannich base hydrochlorides **2a–d**. Imidazole was alkylated with the appropriate Mannich base **2a**–**d** to give ketones **3a**–**d** in good yields (Scheme 1).

*Reagents and conditions:* (i) HN(CH_3_)_2_.HCl, (CH_2_O)_n_, conc. HCl, ethanol, reflux, 2 h; (ii) imidazole, water, reflux, 5 h.

**Figure 2:**
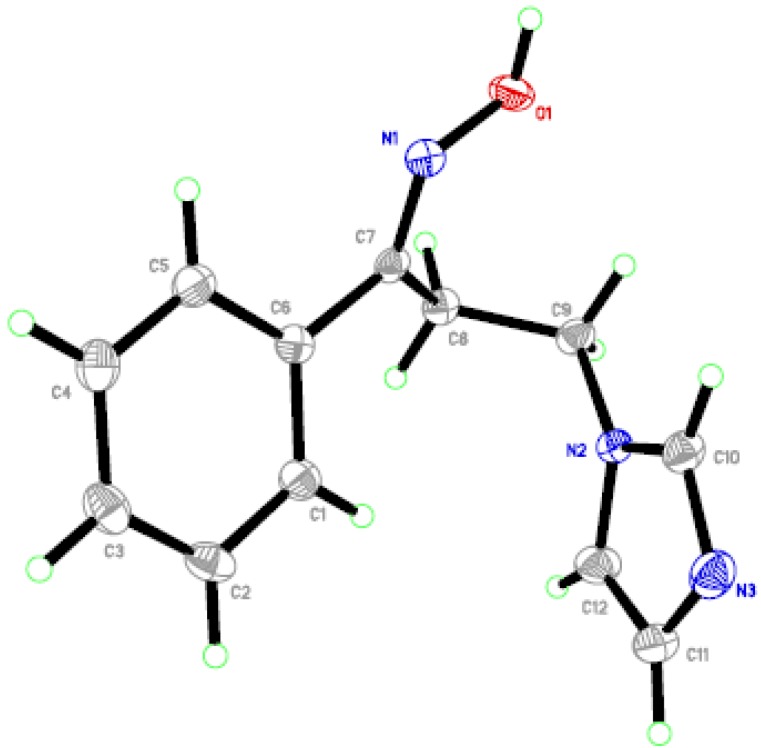
ORTEP diagram of the title compound **4a** drawn at 50% ellipsoids for non-hydrogen atoms.

Ketones **3a**–**d** were allowed to react with hydroxylamine hydrochloride in the presence of potassium hydroxide to yield oximes **4a**–**d**. X-ray crystallography is a decisive analytical tool which can confirm the configuration of the produced oximes **4a**–**d**. Accordingly, the assigned (*E*)-configuration of compounds **4a**–**d** was established *via* single crystal X-ray structure of the oxime **4a** (Figure 2) [17].

**Scheme 2:**
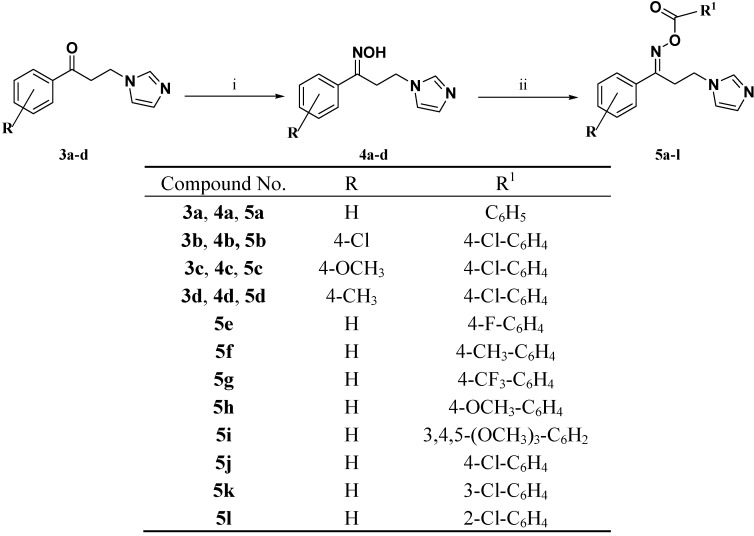
Synthesis of the target compounds **5a**–**l**.

The produced oximes **4a**–**d** were subjected to esterification with the appropriate carboxylic acid derivatives using ethyl-3-(3-dimethylaminopropyl)carbodiimide hydrochloride (EDCI.HCl) in the presence of 4-dimethylaminopyridine (DMAP) to yield the target compounds **5a**–**l** (Scheme 2). The chemical structures of oximes **4a**–**d** and the title compounds **5a**–**l** were confirmed *via* IR, ^1^H-NMR, ^13^C-NMR and mass spectral data.

*Reagents and conditions:* (i) H_2_NOH·HCl, KOH, ethanol, reflux, 18 h.; (ii) an appropriate carboxylic acid, EDCI·HCl, DMAP, DCM, rt, 18 h.

### 2.2. In Vitro Anti-Candida Activity and SARs

Fluconazole is the gold standard azole antifungal and used clinically as the first line of treatment for fungal infections, especially those caused by *C. albicans*. However, its extensive medical use has led to the emergence of resistance [18]. The *in vitro* anti-*Candida* activity of the synthesized imidazole-containing oximes **4a**–**d** and their respective aromatic esters **5a**–**l** was evaluated against two clinical isolates of *Candida*, *C. albicans* and *C. tropicalis,* which are resistant to fluconazole (MIC > 1.6325 µmol/mL). The test compounds **4a**–**d** and **5a**–**l** incorporate a three-carbon atom bridge between the imidazole pharmacophore and the aromatic moiety to gain insight about anti-*Candida* activity of this type of compounds. The anti-*Candida* activities, expressed as diameter of the inhibition zone (DIZ) and minimum inhibition concentration (MIC) for the oximes **4a**–**d**, the target compounds **5a**–**l** as well as for the reference drugs fluconazole and miconazole, are summarized in Table 1.

**Table 1:** Anti-*Candida* activity of oximes **4a**–**d** and the target compounds **5a**–**l**against *Candida albicans* and *Candida tropicalis*.

Compound No	*Candida albicans*		*Candida tropicalis*		
	DIZ ± SD *	MIC (µmol/mL) **		DIZ ± SD *	MIC (µmol/mL) **
**4a**	21 ± 1.0	0.5807		20 ± 0.5	0.5807
**4b**	13 ± 0.6	0.5019		8 ± 1.0	0.5019
**4c**	9 ± 1.15	0.5099		8 ± 1.0	0.2549
**4d**	8 ± 1.0	0.5456		8 ± 1.0	0.5456
**5a**	11 ± 1.2	0.3919		8 ± 1.0	0.7837
**5b**	18 ± 1.1	0.0805		12 ± 1.0	0.6439
**5c**	16 ± 0.4	0.3257		16 ± 1.2	0.3257
**5d**	18 ± 0.9	0.1699		14 ± 0.5	0.3398
**5e**	16 ± 0.9	0.3708		14 ± 0.6	0.3708
**5f**	7 ± 1.0	0.3752		8 ± 1.0	0.1876
**5g**	13 ± 0.6	0.6454		14 ± 1.0	0.3227
**5h**	24 ± 1.1	0.0112		17 ± 1.0	0.3582
**5i**	17 ± 1.1	0.3053		14 ± 0.5	0.3053
**5j**	25 ± 1.0	0.0054		25 ± 1.2	0.1767
**5k**	20 ± 0.9	0.0221		14 ± 0.5	0.7069
**5l**	12 ± 1.0	0.7069		12 ± 0.7	0.3535
Fluconazole	15 ± 0.5	>1.6325		16 ± 0.5	>1.6325
Miconazole	38 ± 1.1	0.0188		24 ± 0.5	0.0024

* The arithmetic mean of the inhibition zone diameters in mean ± standard deviation in mm. ** The lowest concentration of the compound that produced 80% microbial growth inhibition (μmol/mL).

The preliminary anti-*Candida* potential of the test compounds **4a**–**d** and **5a**–**l** was evaluated using the DIZ assay and the results are presented in Table 1. The test compounds displayed a promising anti-*Candida* activity (DIZ = 7–25 mm) where compound **5j** was the most active congener (DIZ = 25 ± 1 and 25 ± 1.2 mm against *C*. *albicans* and *C*. *tropicalis*, respectively).

The oxime **4a** exhibited good anti-*Candida* activity (MIC value = 0.5807 µmol/mL) toward both *C. albicans* and *C. tropicalis*, being more potent than fluconazole (MIC value > 1.6325 µmol/mL) but weaker than miconazole (MIC value = 0.0188 and 0.0024 µmol/mL for *C. albicans* and *C. tropicalis*, respectively). Substitution of the aromatic ring of **4a** with substituents endowed with different electronic and steric properties like chloro, methoxy and/or methyl groups gave compounds **4b**, **4c** and **4d**, respectively, aiming to enhance its anti-*Candida* activity. Unfortunately, the anti-*Candida* activity of **4a** did not improve significantly, except for compound **4c** toward *C. tropicalis* (MIC value = 0.2549 µmol/mL).

De Vita *et al.* reported that the presence of a second aromatic ring could enhance the antifungal activity of azoles [19]. Consequently, the respective aromatic esters **5a**–**l** of the oximes **4a**–**d** were prepared and biologically evaluated as new anti-*Candida* agents. Esterification of the hydroxyl group of **4a** with benzoic acid gave compound **5a** which displayed better anti-*Candida* activity (MIC value = 0.3919 µmol/mL) than that of **4a** toward *C. albicans*. Moreover, esterification of the hydroxyl group of the oximes **4b**–**d** with 4-chlorobenzoic acid gave the respective esters **5b**–**d**. Compounds **5b**–**d** showed better anti-*Candida* profile than their respective oximes **4b**–**d**, where compound **5b** is the most active congener with a MIC value of 0.0805 µmol/mL toward *C. albicans*.

Substitution of the aromatic ester functionality of **5a** with fluoride, methyl and/or trifluoromethyl groups gave compounds **5e**–**g** which exhibited anti*-Candida albicans* activity comparable with that of **5a**, except for compound **5g** (MIC value = 0.6454 µmol/mL) which was weaker than **5a**. Compounds **5e**–**g** displayed better anti-*Candida tropicalis* profiles than that of **5a** where compound **5f** is the most active candidate with a MIC value of 0.1876 µmol/mL.

Substitution of the aromatic ester functionality of **5a** with a group endowed with negative inductive effect and positive mesomeric properties like a methoxy group gave compound **5h**, which showed a comparable anti-*Candida albicans* profile (MIC value = 0.0112 µmol/mL) with that of miconazole (MIC value = 0.0188 µmol/mL) and was about 145-fold more potent than fluconazole (MIC > 1.6325 µmol/mL). This result encouraged us to synthesize the trimethoxy analogue of **5a**, compound **5i**. Unfortunately, **5i** exhibited weaker anti-*Candida albicans* (MIC value = 0.3053 µmol/mL) than that of **5h**.

Compound **5j** emerged as the most active congener in the whole series of synthesized compounds against both *C. albicans* and *C. tropicalis*. Compound **5j**, the 4-chloro analogue of **5a**, exhibited about 3.5-fold and 300-fold more potency than miconazole and the gold standard azole antifungal, fluconazole, respectively, as a drug-like anti-*Candida albicans* agent. The positional isomers of compound **5j**, compounds **5k** and **5l**, displayed weaker anti-*Candida* activity than that of **5j**.

## 3. Experimental

### 3.1. Chemistry

#### 3.1.1. General

Melting points were determined on a Gallenkamp melting point apparatus, and are uncorrected. Infrared (IR) spectra were recorded as KBr disks using the Perkin Elmer FT-IR Spectrum BX apparatus. NMR spectra were carried out on a Bruker NMR spectrometer operating at 500 MHz for ^1^H and 125.76 MHz for ^13^C at the Research Center, College of Pharmacy, King Saud University, Saudi Arabia. TMS was used as internal standard and chemical shift values were recorded in ppm on δ scale. The ^1^H-NMR data were represented as follows: chemical shifts, multiplicity (s. singlet, d. doublet, t. triplet, m. multiplet, br. broad) and number of protons. The ^13^C-NMR data were represented as chemical shifts and type of carbon. Mass spectra were measured on Agilent Triple Quadrupole 6410 QQQ LC/MS with an electrospray ionization (ESI) source. Silica gel thin layer chromatography (TLC) plates from Merck (silica gel precoated aluminium plates with a 245 nm fluorescent indicator) were used for thin layer chromatography. Visualization was performed by illumination with UV light source (254 nm). Column chromatography was carried out on silica gel 60 (0.063–0.200 mm) obtained from Merck and chloroform/methanol (9:0.5) was used as a solvent system.

#### 3.1.2. General Procedure for Preparation of the Ketones **3a**–**d**

The appropriate acetophenone **1a**–**d** (200 mmol), dimethylamine hydrochloride (270 mmol) and paraformaldehyde (90 mmol) were heated to reflux in absolute ethanol (35 mL) in the presence of catalytic amount of concentrated hydrochloric acid (0.5 mL). Reflux of the reaction mixture was continued under stirring for two hours, cooled and acetone (200 mL) was added. The formed Mannich base hydrochlorides **2a**–**d** were precipitated, filtered off and dried. Subsequently, compounds **2a**–**d** (100 mmol) were dissolved in water (100 mL) and imidazole (200 mmol) was added. The reaction mixture was heated to reflux for five hours, cooled and the precipitated solids were collected by filtration to give ketones **3a**–**d** which were pure enough to be used in the next step.

*3-(1H-Imidazol-1-yl)-1-phenylpropan-1-one* (**3a**). Synthesis of **3a** was previously reported [14].

*1-(4-Chlorophenyl)-3-(1H-imidazol-1-yl)propan-1-one* (**3b**). Synthesis of **3b** was previously reported [15].

*3-(1H-Imidazol-1-yl)-1-(4-methoxyphenyl)propan-1-one* (**3c**). Synthesis of **3c** was previously reported [20].

*3-(1H-Imidazol-1-yl)-1-(4-methylphenyl)propan-1-one* (**3d**). Synthesis of **3d** was previously reported [21].

#### 3.1.3. General Procedure for Preparation of the Oximes **4a**–**d**

A mixture of the appropriate ketone **3a**–**d** (10 mmol), hydroxylamine hydrochloride (20 mmol), and KOH (20 mmol) in ethanol (10 mL) was heated to reflux under stirring for 18 h. The reaction mixture was allowed to cool to room temperature and the insoluble solids were filtered off. The filtrate was concentrated under vacuum and the residue was poured onto ice-cold water (15 mL). The precipitated solids were collected by filtration and dried to give oximes **4a**–**d** which were subsequently subjected to the esterification step without any further purification.

*(1E)-N-Hydroxy-3-(1H-imidazol-1-yl)-1-phenylpropan-1-imine* (**4a**). [17] Yield 70%; colourless solid mp. 155–157 °C (ethanol); IR (KBr): *ν* (cm^−1^) 3508 (OH), 3149, 3002, 2703, 1644 (C=N), 1600, 1573, 1221, 758; ^1^H-NMR (CDCl_3_): δ 3.31 (t, *J* = 7.1 Hz, 2H, -C*H_2_*-CH_2_-N), 4.28 (t, *J* = 7.1 Hz, 2H, -CH_2_-C*H_2_*-N), 6.96 (s, 1H, -N-C*H*=CH-N=), 7.07 (s, 1H, -N-CH=C*H*-N=), 7.29–7.49 (m 5H, Ar-H), 7.58 (s, 1H, -N-C*H*=N-); ^13^C-NMR (CDCl_3_): δ 28.3 (-*C*H_2_-CH_2_-N), 41.8 (-CH_2_-*C*H_2_-N), 119.1 (-N-*C*H=CH-N=), 126.1, 128.8, 128.9 (-N-CH=*C*H-N=, Ar-CH), 135.1, 137.0 (Ar-C), 139.5 (-N-*C*H=N-), 155.4 (C=N-OH); MS *m/z* (ESI): 216.0 [M + 1]^+^.

*(1E)-1-(4-Chlorophenyl)-N-hydroxy-3-(1H-imidazol-1-yl)propan-1-imine* (**4b**). The synthesis and characterization of **4b** were previously reported [22].

*(1E)-N-Hydroxy-3-(1H-imidazol-1-yl)-1-(4-methoxyphenyl)propan-1-imine* (**4c**). Yield 65%; pale yellow solid mp. 136–138 °C (ethanol); IR (KBr): *ν* (cm^−1^) 3512 (OH), 3135, 3026, 2632, 1648 (C=N), 1680, 1566, 1228, 752; ^1^H-NMR (CDCl_3_): δ 3.26 (t, *J* = 6.5 Hz, 2H, -C*H_2_*-CH_2_-N), 3.83 (s, 3H, OC*H_3_*), 4.28 (t, *J* = 7.1 Hz, 2H, -CH_2_-C*H_2_*-N), 6.89 (d, *J* = 7.5 Hz, 2H, Ar-H), 6.97 (s, 1H, -N-C*H*=CH-N=), 7.08 (s, 1H, -N-CH=C*H*-N=), 7.44 (d, *J* = 7.5 Hz, 2H, Ar-H), 7.58 (s, 1H, -N-C*H*=N-); ^13^C-NMR (CDCl_3_): δ 28.9 (-*C*H_2_-CH_2_-N), 43.6 (-CH_2_-*C*H_2_-N), 55.4 (O*C*H_3_), 114.0 (Ar-CH), 119.1 (-N-*C*H=CH-N=), 127.4, 127.9, 129.1 (-N-CH=*C*H-N=, Ar-CH, Ar-C), 137.1 (-N-*C*H=N-), 155.4 (C=N-OH), 160.5 (Ar-C); MS *m/z* (ESI): 246.0 [M + 1]^+^.

*(1E)-N-Hydroxy-3-(1H-imidazol-1-yl)-1-(4-methylphenyl)propan-1-imine* (**4d**). Yield 65%; white solid mp. 147–149 °C (ethanol); IR (KBr): *ν* (cm^−1^) 3509 (OH), 3119, 2702, 1639 (C=N), 1679, 1512, 1230, 738; ^1^H-NMR (CDCl_3_): δ 2.27 (s, 3H, C*H_3_*), 3.18 (t, *J* = 7.0 Hz, 2H, -C*H_2_*-CH_2_-N), 4.18 (t, *J* = 7.0 Hz, 2H, -CH_2_-C*H_2_*-N), 6.88 (s, 1H, -N-C*H*=CH-N=), 6.99 (s, 1H, -N-CH=C*H*-N=), 7.08 (d, *J* = 7.8 Hz, 2H, Ar-H), 7.30 (d, *J* = 7.9 Hz, 2H, Ar-H), 7.49 (s, 1H, -N-C*H*=N-); ^13^C-NMR (CDCl_3_): δ 21.3 (*C*H_3_), 28.9 (-*C*H_2_-CH_2_-N), 43.6 (-CH_2_-*C*H_2_-N), 119.1 (-N-*C*H=CH-N=), 125.9, 129.1, 129.4 (-N-CH=*C*H-N=, Ar-CH), 132.6, 137.1, 139.4 (-N-*C*H=N-, Ar-C), 154.7 (C=N-OH); MS *m/z* (ESI): 230.0 [M + 1]^+^.

#### 3.1.4. General Procedure for the Synthesis of the Target Oxime Esters **5a**–**l**

A solution of the appropriate carboxylic acid (7 mmol) and EDCI·HCl (7.3 mmol) was stirred in DCM (75 mL) in the presence of DMAP (400 mg). The appropriate oxime **4a**–**d** (6.9 mmol) was added to the stirred reaction mixture and stirring was continued for further 18 h at room temperature. The reaction mixture was washed successively with water (2 × 20 mL), 10% NaHCO_3_ solution (2 × 15 mL), and water (2 × 15 mL). The organic layer was separated, dried (Na_2_SO_4_) and evaporated under reduced pressure and the residue was purified either by recrystallisation (for solids) or by column chromatography (for oils).

*(E)-3-(1H-Imidazol-1-yl)-1-phenylpropan-1-one O-benzoyl oxime* (**5a**). Yield 41%; colourless viscous oil; IR (KBr): ν (cm^−1^) 3115, 2943, 1746 (C=O), 1650 (C=N), 1510, 1243, 735; ^1^H-NMR (CDCl_3_): δ (ppm) = 3.38 (t, *J* = 7.1 Hz, 2H, -C*H_2_*-CH_2_-N), 4.21 (t, *J* = 7.1 Hz, 2H, -CH_2_-C*H_2_*-N), 6.84 (s, 1H,-N-C*H*=CH-N=), 6.95 (s, 1H, -N-CH=C*H*-N=), 7.36-7.46 (m, 6H, -N-C*H*=N-, Ar-H), 7.55-7.60 (m, 2H, Ar-H), 7.96 (d, *J* = 7.6 Hz, 2H, Ar-H_._); ^13^C-NMR (CDCl_3_): δ 31.0 (-*C*H_2_-CH_2_-N), 43.7 (-CH_2_-*C*H_2_-N), 118.8 (-N-*CH*=CH-N=), 127.3, 128.6, 128.8, 129.1, 129.6, 130.0, 131.3 (-N-CH=*C*H-N=, Ar-CH, Ar-C), 133.0, 133.8, 136.9 (-N-*C*H=N-, Ar-CH, Ar-C), 163.4 (C=N), 163.5 (C=O); MS *m/z* (ESI): 320.1 [M + 1]^+^.

*(E)-1-(4-Chlorophenyl)-3-(1H-imidazol-1-yl)propan-1-one O-4-chlorobenzoyl oxime* (**5b**). Yield 56%; white solid mp. 132–134 °C (isopropanol); IR (KBr): ν (cm^−1^) 3107, 1744 (C=O), 1650 (C=N), 1560, 1513, 1261, 748; ^1^H-NMR (CDCl_3_): δ (ppm) = 3.43 (t, *J* = 6.9 Hz, 2H, -C*H_2_*-CH_2_-N), 4.29 (t, *J* = 6.9 Hz, 2H, -CH_2_-C*H_2_*-N), 6.89 (s, 1H, -N-C*H*=CH-N=), 7.02 (s, 1H, -N-CH=C*H*-N=), 7.41 (d, *J* = 8.5 Hz, 2H, Ar-H_._), 7.49–7.51 (m, 3H, -N-C*H*=N-, Ar-H), 7.60 (d, *J* = 8.5 Hz, 2H, Ar-H_._), 7.94 (d, *J* = 8.5 Hz, 2H, Ar-H); ^13^C-NMR (CDCl_3_): δ 30.8 (-*C*H_2_-CH_2_-N), 43.7 (-CH_2_-*C*H_2_-N), 118.8 (-N-*CH*=CH-N=), 126.9, 128.5, 129.2, 129.4, 130.0, 130.9, 131.2, (-N-CH=*C*H-N=, Ar-CH, Ar-C), 136.9, 137.8, 140.4 (-N-*C*H=N-, Ar-C), 162.5 (C=N), 162.6 (C=O); MS *m/z* (ESI): 388.0 [M ]^+^.

*(E)-3-(1H-Imidazol-1-yl)-1-(4-methoxyphenyl)propan-1-one O-4-chlorobenzoyl oxime* (**5c**). Yield 70%; white solid mp. 131–133 °C (isopropanol); IR (KBr): ν (cm^−1^) 3123, 2366, 1758 (C=O), 1684 (C=N), 1564, 1514, 1252, 747; ^1^H-NMR (CDCl_3_): δ (ppm) = 3.44 (t, *J* = 6.9 Hz, 2H, -C*H_2_*-CH_2_-N), 3.87 (OC*H_3_*), 4.32 (t, *J* = 7.0 Hz, 2H, -CH_2_-C*H_2_*-N), 6.92 (s, 1H, -N-C*H*=CH-N=), 6.96 (d, *J* = 8.8 Hz, 2H, Ar-H_._), 7.09 (s, 1H, -N-CH=C*H*-N=), 7.49 (d, *J* = 8.6 Hz, 2H, Ar-H_._), 7.67 (d, *J* = 9.0 Hz, 2H, Ar-H_._), 7.77 (s, 1H, -N-C*H*=N-), 7.94 (d, *J* = 8.6 Hz, 2H, Ar-H); ^13^C-NMR (CDCl_3_): δ 30.5 (-*C*H_2_-CH_2_-N), 44.2 (-CH_2_-*C*H_2_-N), 55.5 (O*C*H_3_), 114.5 (Ar-CH), 119.1 (-N-*CH*=CH-N=), 128.5, 128.9, 129.2, 130.9, 131.2, (-N-CH=*C*H-N=, Ar-CH, Ar-C), 136.8, 140.2 (-N-*C*H=N-, Ar-C), 162.3, 162.8, 162.9 (C=N, C=O, Ar-C); MS *m/z* (ESI): 384.2 [M + 1]^+^

*(E)-3-(1H-Imidazol-1-yl)-1-(4-methylphenyl)propan-1-one O-4-chlorobenzoyl oxime* (**5d**). Yield 58%; white solid mp. 142–144 °C (isopropanol); IR (KBr): ν (cm^−1^) 3065, 1744 (C=O), 1654 (C=N), 1646, 1559, 1508, 1254, 749; ^1^H-NMR (CDCl_3_): δ (ppm) = 2.42 (s, 3H, C*H_3_*), 3.46 (t, *J* = 6.9 Hz, 2H, -C*H_2_*-CH_2_-N), 4.31 (t, *J* = 6.9 Hz, 2H, -CH_2_-C*H_2_*-N), 6.91 (s, 1H, -N-C*H*=CH-N=), 7.09 (s, 1H, -N-CH=C*H*-N=), 7.27 (d, *J* = 7.9 Hz, 2H, Ar-H_._), 7.49 (d, *J* = 8.5 Hz, 2H, Ar-H_._), 7.62 (d, *J* = 8.0 Hz, 2H, Ar-H_._), 7.79 (s, 1H, -N-C*H*=N-), 7.94 (d, *J* = 8.5 Hz, 2H, Ar-H); ^13^C-NMR (CDCl_3_): δ 21.5 (*C*H_3_), 30.6 (-*C*H_2_-CH_2_-N), 44.2 (-CH_2_-*C*H_2_-N), 119.1 (-N-*CH*=CH-N=), 127.2, 128.4, 128.5, 129.2, 129.9, 130.9, 131.2, (-N-CH=*C*H-N=, Ar-CH, Ar-C), 136.8, 140.2, 142.1 (-N-*C*H=N-, Ar-C_._), 162.7 (C=N), 163.3 (C=O); MS *m/z* (ESI): 368.2 [M + 1]^+^.

*(E)-3-(1H-Imidazol-1-yl)-1-phenylpropan-1-one O-4-fluorobenzoyl oxime* (**5e**). Yield 62%; pale yellow solid mp. 114–116 °C (isopropanol); IR (KBr): ν (cm^−1^) 3115, 2848, 1746 (C=O), 1660 (C=N), 1571, 1249, 739; ^1^H-NMR (CDCl_3_): δ (ppm) = 3.46 (t, *J* = 7.0 Hz, 2H, -C*H_2_*-CH_2_-N), 4.29 (t, *J* = 7.0 Hz, 2H, -CH_2_-C*H_2_*-N), 6.91 (s, 1H, -N-C*H*=CH-N=), 7.02 (s, 1H, -N-CH=C*H*-N=), 7.18–7.21 (m, 2H, Ar-H), 7.43–7.52 (m, 4H, -N-C*H*=N-, Ar-H), 7.68 (d, *J* = 7.4 Hz, 2H, Ar-H_._), 8.03–8.05 (m, 2H, Ar-H); ^3^C-NMR (CDCl_3_): δ 30.9 (-*C*H_2_-CH_2_-N), 43.8 (-CH_2_-*C*H_2_-N), 116.1 (d, *J* = 22.1 Hz, Ar-CH), 118.8 (-N-*CH*=CH-N=), 124.9, (d, *J* = 2.6 Hz, Ar-C), 127.3, 129.1, 129.8, 131.4 (-N-CH=*C*H-N=, Ar-CH), 132.2 (d, *J* = 9.4 Hz, Ar-CH), 132.8, 136.9 (-N-*C*H=N-, Ar-C), 162.5 (C=N), 163.5 (C=O), 167.1 (d, *J* = 254.0 Hz, Ar-C); MS *m/z* (ESI): 338.2 [M + 1]^+^.

*(E)-3-(1H-Imidazol-1-yl)-1-phenylpropan-1-one O-4-methylbenzoyl oxime* (**5f**). Yield 62%; pale yellow solid mp. 125–127 °C (isopropanol); IR (KBr): ν (cm^−1^) 3115, 2964, 1736 (C=O), 1647 (C=N), 1605, 1506, 1248, 750; ^1^H-NMR (CDCl_3_): δ (ppm) = 2.37 (s, 3H, C*H_3_*), 3.36 (t, 2H, *J* = 7.1 Hz, -C*H_2_*-CH_2_-N), 4.21 (t, 2H, *J* = 7.0 Hz -CH_2_-C*H_2_*-N), 6.84 (s, 1H, -N-C*H*=CH-N=), 6.94 (s, 1H, -N-CH=C*H*-N=), 7.23 (d, *J* = 7.8 Hz, 2H, Ar-H), 7.35–7.41 (m, 4H, -N-C*H*=N-, Ar-H), 7.59 (d, *J* = 7.0 Hz, 2H, Ar-H_._), 7.85 (d, *J* = 8.0 Hz, 2H, Ar-H); ^13^C-NMR (CDCl_3_): δ 21.8 (*C*H_3_), 31.1 (-*C*H_2_-CH_2_-N), 43.7 (-CH_2_-*C*H_2_-N), 118.8 (-N-*CH*=CH-N=), 125.8, 127.2, 129.0, 129.5, 129.6, 130.1, 131.2, 133.1 (-N-CH=*C*H-N=, Ar-CH, Ar-C), 136.9 (-N-*C*H=N-), 144.7 (Ar-C), 163.2 (C=N), 163.5 (C=O); MS *m/z* (ESI): 334.0 [M + 1]^+^.

*(E)-3-(1H-Imidazol-1-yl)-1-phenylpropan-1-one O-4-(trifluoromethyl)benzoyl oxime* (**5g**). Yield 39%; white solid mp. 125–127 °C (isopropanol); IR (KBr): ν (cm^−1^) 3050, 2360, 1750 (C=O), 1653 (C=N), 1559, 1507, 1264, 737; ^1^H-NMR (CDCl_3_): δ (ppm) = 3.47–3.49 (m, 2H, -C*H_2_*-CH_2_-N), 4.29–4.32 (m, 2H, -CH_2_-C*H_2_*-N), 6.91 (s, 1H, -N-C*H*=CH-N=), 7.05 (s, 1H, -N-CH=C*H*-N=), 7.36–7.55 (m, 4H, -N-C*H*=N-, Ar-H), 7.71–7.72 (m, 2H, Ar-H), 7.79 (d, *J* = 8.0 Hz, 2H, Ar-H_._), 8.13 (d, *J* = 8.0 Hz, 2H, Ar-H); ^13^C-NMR (CDCl_3_): δ 30.8 (-*C*H_2_-CH_2_-N), 43.8 (-CH_2_-*C*H_2_-N), 118.8 (-N-*CH*=CH-N=), 125.8 (d, *J* = 3.4 Hz, *C*F_3_), 125.9, 127.3, 128.6, 129.2, 129.8, 130.0, 131.6, 132.6 (-N-CH=*C*H-N=, Ar-CH, Ar-C), 135.6, 136.9 (-N-*C*H=N-, Ar-C), 162.4 (C=N), 164.0 (C=O); MS *m/z* (ESI): 388.1 [M + 1]^+^.

*(E)-3-(1H-Imidazol-1-yl)-1-phenylpropan-1-one O-4-methoxybenzoyl oxime* (**5h**). Yield 40%; off white solid mp. 108–110 °C (isopropanol); IR (KBr): ν (cm^−1^) 3117, 2968, 1735 (C=O), 1650 (C=N), 1602, 1507, 1248, 765; ^1^H-NMR (CDCl_3_): δ (ppm) = 3.46 (t, *J* = 6.9 Hz, 2H, -C*H_2_*-CH_2_-N), 3.91 (s, 3H, OC*H_3_*), 4.30 (t, *J* = 6.9 Hz, 2H, -CH_2_-C*H_2_*-N), 6.94 (s, 1H, -N-C*H*=CH-N=), 7.01 (d, *J* = 8.8 Hz, 2H, Ar-H), 7.10 (s, 1H, -N-CH=C*H*-N=), 7.45–7.50 (m, 4H, -N-C*H*=N-, Ar-H), 7.67 (d, *J* = 7.0 Hz, 2H, Ar-H.), 8.01 (d, *J* = 8.8 Hz, 2H, Ar-H); ^13^C-NMR (CDCl_3_): δ 31.0 (-*C*H_2_-CH_2_-N), 43.7 (-CH_2_-*C*H_2_-N), 55.6 (O*C*H_3_), 114.1 (Ar-*C*H), 118.8 (-N-*CH*=CH-N=), 120.7, 127.2, 129.0, 130.0, 131.2, 131.7, 133.1 (-N-CH=*C*H-N=, Ar-CH, Ar-C), 136.9 (-N-*C*H=N-), 162.9 (C=N), 163.2 (Ar-C), 164.0 (C=O); MS *m/z* (ESI): 350.0 [M + 1]^+^.

*(E)-3-(1H-Imidazol-1-yl)-1-phenylpropan-1-one O-3,4,5-trimethoxybenzoyl oxime* (**5i**). Yield 53%; white solid mp. 135–137 °C (isopropanol); IR (KBr): ν (cm^−1^) 3103, 2938, 1742 (C=O), 1645 (C=N), 1593, 1503, 1231, 749; ^1^H-NMR (CDCl_3_): δ (ppm) = 3.45 (t, *J* = 6.8 Hz, 2H, -C*H_2_*-CH_2_-N), 3.92 (s, 6H, 2 × OC*H_3_*), 3.94 (s, 3H, OC*H_3_*), 4.28 (t, *J* = 6.8 Hz, 2H, -CH_2_-C*H_2_*-N), 6.91 (s, 1H, -N-C*H*=CH-N=), 7.02 (s, 1H, -N-CH=C*H*-N=), 7.27–7.50 (m, 6H, -N-C*H*=N-, Ar-H), 7.67 (d, *J* = 7.0 Hz, 2H, Ar-H_._);^13^C-NMR (CDCl_3_): δ 30.9 (-*C*H_2_-CH_2_-N), 43.6 (-CH_2_-*C*H_2_-N), 56.5 (2 × O*C*H_3_), 61.0 (O*C*H_3_), 106.9 (Ar-*C*H), 118.6 (-N-*CH*=CH-N=), 123.5, 127.3, 129.1, 130.1, 131.3, 132.9 (-N-CH=*C*H-N=, Ar-CH, Ar-C), 136.8 (-N-*C*H=N-), 142.9, 153.2 (Ar-C), 163.2 (C=N), 163.6 (C=O); MS *m/z* (ESI): 410.1 [M + 1]^+^.

*(E)-3-(1H-Imidazol-1-yl)-1-phenylpropan-1-one O-4-chlorobenzoyl oxime* (**5j**). Yield 54%; colourless crystals mp. 126–128 °C (isopropanol); IR (KBr): ν (cm^−1^) 3115, 2970, 1743 (C=O), 1648 (C=N), 1508, 1249, 736; ^1^H-NMR (CDCl_3_): δ (ppm) = 3.44 (t, *J* = 6.7 Hz, 2H, -C*H_2_*-CH_2_-N), 4.27 (t, *J* = 6.7 Hz, 2H, -CH_2_-C*H_2_*-N), 6.90 (s, 1H, -N-C*H*=CH-N=), 7.02 (s, 1H, -N-CH=C*H*-N=), 7.45–7.50 (m, 6H, -N-C*H*=N-, Ar-H), 7.68 (d, *J* = 8.4 Hz, 2H, Ar-H_._), 7.95 (d, *J* = 8.4 Hz, 2H, Ar-H); ^13^C-NMR (CDCl_3_): δ 30.9 (-*C*H_2_-CH_2_-N), 43.7 (-CH_2_-*C*H_2_-N), 118.7 (-N-*CH*=CH-N=), 127.1, 127.3, 129.1, 129.2, 130.1, 130.9, 131.4, 132.8 (-N-CH=*C*H-N=, Ar-CH, Ar-C), 136.9 (-N-*C*H=N-), 140.2 (Ar-C_._), 162.7 (C=N), 163.7 (C=O); MS *m/z* (ESI): 354.1 [M + 1]^+^.

*(E)-3-(1H-Imidazol-1-yl)-1-phenylpropan-1-one O-3-chlorobenzoyl oxime* (**5k**). Yield 61%; pale yellow viscous oil; IR (KBr): ν (cm^−1^) 3113, 1751 (C=O), 1654 (C=N), 1510, 1282, 739; ^1^H-NMR (DMSO-*d_6_*): δ (ppm) = 3.52 (br. s, 2H, -C*H_2_*-CH_2_-N), 4.30 (br. s, 2H, -CH_2_-C*H_2_*-N), 6.80 (s, 1H, -N-C*H*=CH-N=), 7.17 (s, 1H, -N-CH=C*H*-N=), 7.51–7.82 (m, 8H, -N-C*H*=N-, Ar-H), 8.00 (d, *J* = 1.5 Hz, 2H, Ar-H_._); ^13^C-NMR (DMSO-*d_6_*): δ 30.1 (-*C*H_2_-CH_2_-N), 43.0 (-CH_2_-*C*H_2_-N), 119.3 (-N-*CH*=CH-N=), 127.3, 128.1, 128.5, 128.9, 130.3, 130.9, 131.1, 132.9, 133.6, 133.7 (-N-CH=*C*H-N=, Ar-CH, Ar-C), 137.1 (-N-*C*H=N-), 161.7 (C=N), 164.8 (C=O); MS *m/z* (ESI): 354.1 [M]^+^.

*(E)-3-(1H-Imidazol-1-yl)-1-phenylpropan-1-one O-2-chlorobenzoyl oxime* (**5l**). Yield 60%; white solid mp. 118–120 °C (isopropanol); IR (KBr): ν (cm^−1^) 3054, 1763 (C=O), 1658 (C=N), 1640, 1511, 1265, 739; ^1^H-NMR (DMSO-*d_6_*): δ (ppm) = 3.45 (br. s, 2H, -C*H_2_*-CH_2_-N), 4.25 (br. s, 2H, -CH_2_-C*H_2_*-N), 6.80 (s, 1H, -N-C*H*=CH-N=), 7.08 (s, 1H, -N-CH=C*H*-N=), 7.51-7.67 (m, 7H, -N-C*H*=N-, Ar-H), 7.75 (d, *J* = 6.9 Hz, 2H, Ar-H_._), 7.90 (d, *J* = 7.2 Hz, 1H, Ar-H_._); ^13^C-NMR (DMSO-*d_6_*): δ 30.2 (-*C*H_2_-CH_2_-N), 43.0 (-CH_2_-*C*H_2_-N), 119.2 (-N-*CH*=CH-N=), 127.3, 127.6, 128.5, 128.8, 128.9, 130.9, 131.1, 131.3, 131.9, 132.8, 133.7 (-N-CH=*C*H-N=, Ar-CH, Ar-C), 137.1 (-N-*C*H=N-), 162.2 (C=N), 164.6 (C=O); MS *m/z* (ESI): 354.1 [M]^+^.

### 3.2. Anti-Candida Activity

#### 3.2.1. Anti-*Candida* Agents

Miconazole was purchased from Sigma-Aldrich Co. (St. Louis, MO, USA) and fluconazole from Shouguang-Fukang Pharmaceutical Ltd. (Shandong, China). The antifungal discs (containing 25 µg fluconazole and/or 10 µg miconazole) were purchased from ROSCO (Neo-Sensitabs, Taastrup, Denmark).

Dimethyl sulfoxide (100%) was used to dissolve stock solutions of miconazole, fluconazole and/or the synthesized compounds **4a**–**d** and **5a**–**l** to obtain an initial concentration of 1000 µg/mL. These stock solutions were then diluted to the desired concentration with sterile distilled water. Miconazole and fluconazole antifungal discs were stored at –80 °C until used.

#### 3.2.2. Media

Liquid RPMI 1640 medium supplemented with L-glutamine was purchased from Sigma-Aldrich Co. (St. Louis, MO, USA) and was added to 2% sodium bicarbonate and 0.165 M morpholine- propane sulfonic acid (MOPS) from Dojindo Laboratories (Kumamoto, Japan) then adjusted to pH 7.0. Sabouraud Dextrose Agar (SDA) and Brain Heart Infusion Broth (BHI) from Difco Laboratories (Detroit, MI, USA). Potato dextrose agar (PDA) was purchased from Eiken Chemical Co. Ltd. (Tokyo, Japan).

#### 3.2.3. Organisms

Two clinical isolates of *Candida* species were obtained from King Khaled Hospital, Riyadh, Saudi Arabia. One was identified as *C*. *albicans* and the other as *C. tropicalis.* The yeasts were stored at –70 °C in BHI with glycerol 5% until tested.

#### 3.2.4. Preparation of Inocula

Preparation of inocula for the broth microdilution testing was performed in accordance with CLSI documents M27-A2 [23] with RPMI 1640 medium. Yeast isolates were subcultured at 35 °C for 48 h on PDA plates. *Candida* cells were then recovered and suspended in 5 mL of sterile saline. The turbidity of each suspension was adjusted to a 0.5 McFarland standard (corresponding to 1–3 × 10^6^ to 5–3 × 10^6^ CFU/mL) at a wavelength of 530 nm according to the reported method [23]. Each suspension was diluted 1,000-fold with sterile RPMI 1640 medium to give a final inoculum of 1–3 × 10^3^ to 5–3× 10^3^ CFU/mL.

#### 3.2.5. Disk Diffusion Assay

The disk diffusion assay was performed as described previously [24]. Colonies obtained from the *Candida* strains under test were suspended in sterile saline and adjusted to a 0.5 McFarland standard (corresponding to 5 × 10^6^ CFU/mL). An aliquot of 100 µL of each yeast suspension was spread uniformly onto SDA plates. Six mm Whatmann filter paper disks were impregnated with 1000 µg of the synthesized compounds **4a**–**d** and **5a**–**l** and were allowed to dry. Then they were placed onto the surface of the inoculated agar plates together with the standard antifungal discs which were then incubated at 35 °C. Diameters of inhibition zones were measured at 24 h.

#### 3.2.6. Antifungal Susceptibility Studies

The MIC of the reference standards and/or the synthesized compounds **4a**–**d** and **5a**–**l** were determined with a microdilution test (M27-A2 Protocol), according to the reference method of the CLSI. The previously prepared yeast inocula (100 µL) were added to each well of 96-well flat-bottom microdilution plates; each well contained 100 µL of twofold serial dilutions of the standard or the synthesized compounds **4a**–**d** and **5a**–**l** ranging from 1 µg/mL to 500 µg/mL in RPMI 1640 medium. Readings were measured at 490 nm with a microplate ELISA reader after each plate was incubated at 35 °C for 48 h. The MICs for the reference standards and/or the synthesized compounds were determined with 80% growth inhibition at the end point relative to the turbidity of the growth control. 

## 4. Conclusions

Anti-*Candida* activities of certain new imidazole-containing oximes **4a**–**d** and their respective aromatic esters **5a**–**l** have been reported. The synthesized compounds **4a**–**d** and **5a**–**l** exhibited anti-*Candida* activity better than that of the gold standard antifungal drug, fluconazole. Compound **5j** emerged as the most active congener among the all synthesized compounds, being about 3.5-fold and 300-fold more potent than miconazole and fluconazole, respectively. Compound **5j** could be considered as a prodrug and could serve as a new lead for anti-*Candida* agents.
